# Modifying Metastable Sr_1–*x*_BO_3−δ_ (B = Nb, Ta, and Mo) Perovskites for Electrode Materials

**DOI:** 10.1021/acsami.1c05743

**Published:** 2021-06-16

**Authors:** Tochukwu Ofoegbuna, Benjamin Peterson, Natalia da Silva Moura, Roshan Nepal, Orhan Kizilkaya, Carsyn Smith, Rongying Jin, Craig Plaisance, John C. Flake, James A. Dorman

**Affiliations:** † Cain Department of Chemical Engineering, Louisiana State University, Baton Rouge, Louisiana 70803, United States; ‡ Department of Physics and Astronomy, Louisiana State University, Baton Rouge, Louisiana 70803, United States; § Center for Advanced Microstructure Devices, Louisiana State University, Baton Rouge, Louisiana 70803, United States; ∥ St. Joseph’s Academy, Baton Rouge, Louisiana 70803, United States

**Keywords:** defect engineering, insulator-to-metal transitions, enhanced electron transport, electrochemical devices, first-principles calculations

## Abstract

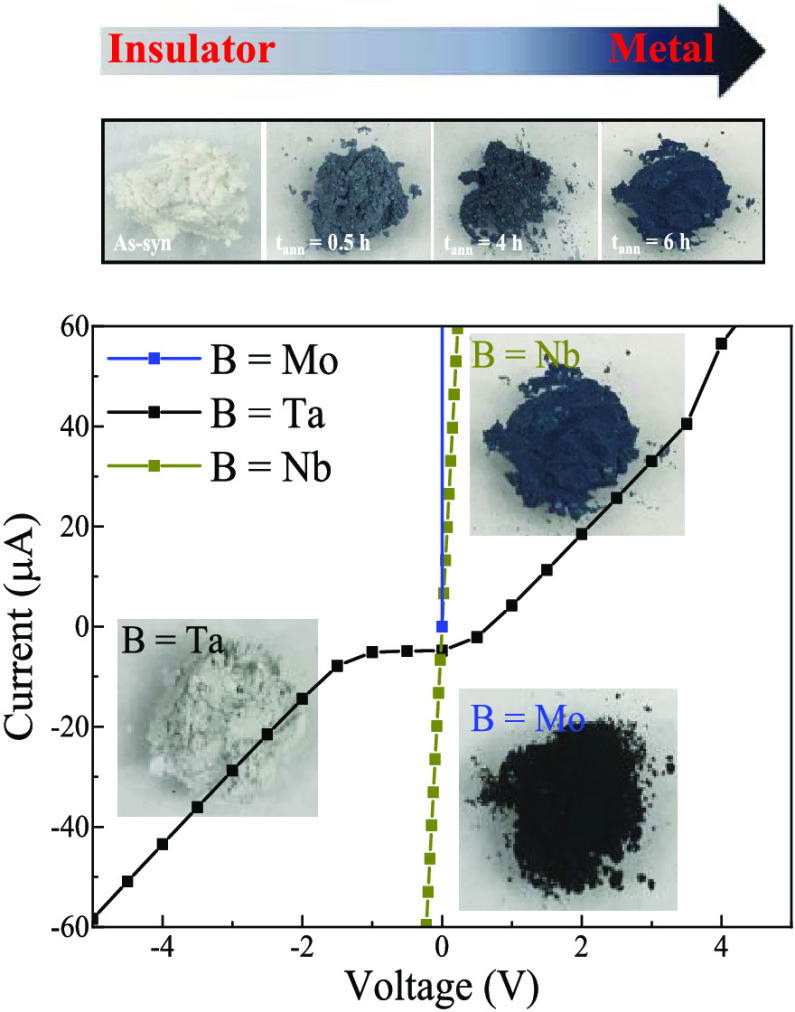

The presence of surface/deep defects in 4d- and 5d-perovskite oxide (ABO_3_, B = Nb, Ta, Mo, etc.) nanoparticles (NPs), originating from multivalent B-site cations, contributes to suppressing their metallic properties. These defect states can be removed using a H_2_/Ar thermal treatment, enabling the recovery of their electronic properties (i.e., low electrical resistivity, high carrier concentration, etc.) as expected from their electronic structure. Therefore, to engineer the electronic properties of these metastable perovskites, an oxygen-controlled crystallization approach coupled with a subsequent H_2_/Ar treatment was utilized. A comprehensive study of the effect of the post-treatment time on the electronic properties of these perovskite NPs was performed using a combination of scattering, spectroscopic, and computational techniques. These measurements revealed that a metallic-like state is stabilized in these oxygen-reduced NPs due to the suppression of deep rather than surface defects. Ultimately, this synthetic approach can be employed to synthesize ABO_3_ perovskite NPs with tunable electronic properties for application into electrochemical devices.

## 1 Introduction

Owing to their metallic responses,^1,2^ 4d- and 5d*-*perovskite oxides (ABO_3_, A = alkaline earth metal and B = Nb, Ta, Mo, etc.) have emerged as attractive charge transport materials for photovoltaic devices.^2^ Recently, it was demonstrated that insulator-to-metal transitions (IMT)^3^ and confinement effects^4^ can be utilized to drive metallic behavior in these materials under ambient conditions. This enables the design of new energy-efficient schemes for electrochemical applications.^5^ While there have been attempts to synthesize nanocrystals of these conductive perovskite oxides using solution-based methods,^1,6^ the resultant insulating nanoparticles (NPs) possess B-site cations that are stabilized in highly oxidized states (i.e., Nb^5+^, Ta^5+^, Mo^6+^, etc.).^7^ These multivalent B-site cations, present as surface/deep defects,^1,8^ limit charge transport by modifying the available free carriers. It is evident from these reports that the wet-chemical synthesis of metastable 4d- and 5d-perovskites with ABO_3_ stoichiometry and possessing metallic responses still remains a synthetic challenge.

Previous studies demonstrated that the IMT can be induced by thermally treating the sample in oxygen-reduced atmospheres.^9,10^ For example, the electrical resistivity of stoichiometric strontium titanate (SrTiO_3_, STO), which is known to be a band insulator,^11^ is drastically reduced to a metallic-like state (∼10^2^ Ω) when thermally treated at 800 °C.^10^ Similarly, a 99% decrease in resistivity (∼10^1^ Ω cm) can be obtained for oxygen-reduced titanium dioxide (TiO_2–*x*_).^12^ The low electrical resistivities observed for these crystals are due to the formation of oxygen vacancies,^10,13^ which generate midgap states that donate free electrons (n-type conductivity). However, in the case of metastable ABO_3_ perovskites, oxygen-reduced environments such as H_2_/Ar inhibit overoxidation of the B-site ion by removing excess oxygen from the crystal lattice.^2,9^ These multivalent B-site ions act as charge carrier traps (surface) and transport barriers (bulk),^9^ motivating the need to prevent their formation during crystallization. As a result of reducing the oxidation state of the B-site ion, the available d states for free carriers dramatically decrease the electrical resistivity (>10^8^ Ω cm for insulators to ∼10^–4^ Ω cm for metals) of these structures.^1^ In essence, the passivation of these surface/deep defect states using thermal reduction initiates an IMT, which enables improved electrochemical^5^ and photocatalytic^9^ performances by enhancing the charge transport. For these reasons, the ability to control the IMT of oxides using H_2_/Ar reduction is an effective approach for designing/developing conductive metal oxides. Therefore, H_2_/Ar annealing is leveraged to engineer the electronic properties of these metastable 4d- and 5d-perovskite NPs to obtain the expected optoelectronic responses by inducing an IMT.

In the present work, Sr_1–*x*_BO_3−δ_ (SBO, B = Nb, Ta, and Mo) NPs were synthesized using an oxygen-controlled wet-chemical synthetic method to form the archetypal perovskite framework. The as-synthesized NPs were annealed in a reducing atmosphere (H_2_/Ar) to induce metallic responses by removing excess oxygen within the crystal lattice. To understand the effect the H_2_/Ar treatment has on the surface and deep defect states of SBO NPs, the samples were systematically treated at various times. The structural, electronic, and chemical properties of these powders were characterized to elucidate the effect of the surface and deep defect state suppression on the overall electrochemical performance. Electrical transport measurements show that even though these materials can be synthesized, their fully metallic nature, with the exception of B = Mo, is lessened to a metallic-like response due to the still present deep defect states. Furthermore, density functional theory (DFT) and Boltzmann transport calculations demonstrate that the n-type (B = Nb and Mo) and p-type (B = Ta) conductions of these NPs are responsible for their transport properties. These results suggest that post-processing as-synthesized SBO NPs in an oxygen-reduced atmosphere can be used to restore their expected electronic properties. Overall, these experimental and computational results on metallic-like SBO NPs not only provide new insights into the understanding of IMT in metal oxides but also contribute to the design of next-generation electrochemical devices.

## 2 Experimental Section

### 2.1 Synthesis of Metastable Perovskite Oxide Nanocrystals (Sr_1–*x*_BO_3_, B = Nb, Ta, and Mo)

Sr_1–*x*_BO_3_ (SBO) nanoparticles were synthesized using a two-step coprecipitation/oxygen-controlled crystallization approach described elsewhere.^7^ Briefly, A-site (Sr(NO_3_)_2_, Alfa Aesar, 99.0%, ACS grade), and B-site (NbCl_5_, Alfa Aesar, 99.0% metals basis; (NH_4_)_6_Mo_7_O_24_·4H_2_O, Sigma-Aldrich; TaCl_5_, Alfa Aesar, 99.99% metals basis) salt precursors were precipitated using ammonium hydroxide (NH_4_OH, 28–30%, ACS grade) at a pH of 9.5. Next, the as-prepared powder was ground with a eutectic molar ratio of NaNO_3_ (high purity grade, VWR Amresco, 99.0%) and KNO_3_ (ACS grade, VWR Amresco) to form a homogeneous powder. The mixture was then transferred to a porcelain boat and heated in a tube furnace at 600 °C under 0.2 Torr pressure for 2 h. After cooling, the resultant powder was washed several times with deionized water and dried overnight at 100 °C. Finally, as-synthesized NPs were annealed in a tube furnace at 800 °C under a H_2_/Ar (5/95%) atmosphere with varying times (0–6 h).

### 2.2 Structural Characterization

The crystal structure of SBO (B = Nb, Ta, and Mo) NPs was identified by performing powder X-ray diffraction (XRD) using PANalytical X-ray diffractometer operating at 45 kV and 40 mA. The θ–2θ radial scan was performed over the range 5–70° with a step size of 0.04° and dwell time of 60 s, using Cu Kα (λ = 1.54 Å) as a radiation source. Rietveld refinement was performed on the resultant diffraction pattern using the GSAS II software^14^ for structural verification. Full structural refinement was achieved by performing the procedure outlined in ref (7). A Perkins Elmer Optima 8000 inductively coupled plasma optical emission spectrometer (ICP-OES) equipped with an autosampler was used to verify the refined stoichiometries. Samples for ICP-OES analyses were prepared by digesting 12.5 mg of SBO NPs in a HNO_3_ (MiliporeSigma, 65%) and HCl (VWR BDH Chemicals, 38%) solution, which is heated to ∼90 °C. Then, the digested sample is diluted to 40 ppm using 2% HNO_3_. Thermogravimetric analysis (TGA)–differential scanning calorimetry (DSC) was performed using a TA SDT Q600 under a H_2_/Ar (5/95%) gas flow to monitor the crystallization process of SBO (B = Nb) powders. The temperature was increased from 25 to 800 °C at a rate of 10 °C/min and subsequently held at 800 °C for 6 h. Iodometric titrations were performed to investigate the oxygen vacancy concentration (δ) in SBO lattices using the procedure presented in ref (7). Nanoparticle sizes and structure were determined by transmission electron microscopy (TEM, JEOL JEM-1400 operating at an accelerating voltage of 120 kV) and high-resolution TEM (HRTEM, JEOL JEM-2011 operating at an accelerating voltage of 200 kV). The powder samples were dispersed in toluene and drop casted on a lacey carbon type-A, 300 mesh copper grid prior to imaging. The corresponding elemental composition was determined using a FEI Quanta 3D FIB microscope equipped with an EDAX Apollo XL EDX detector operating at an accelerating voltage of 20 kV and a current of 4 nA. The working distance was maintained at ∼10 mm. The samples were dried overnight on carbon tape and then sputtered with Pt for 5 min to limit charging.

### 2.3 Optical and Electronic Characterization

The absorption spectra of SBO NPs were recorded using a Perkin-Elmer Lambda 900 UV/vis/NIR spectrometer equipped with an integrating sphere and a center-mounted sample holder. The absorption scans were obtained using a scan rate of 1 nm/s with no monochromator change. The powder samples were dried onto a glass substrate to perform UV–vis measurements. The electronic structures of SBO NPs were measured by performing X-ray photoemission spectroscopy (XPS) measurements at the 5-meter toroidal grating monochromator (TGM) beamline at the Center for Advanced Microstructures and Devices (CAMD) at the Louisiana State University. The beamline and endstation are described in detail elsewhere.^15^ The beamline is equipped with an ultrahigh vacuum chamber endstation maintained at a base pressure of 10^–10^ Torr, a DAR-400 dual Mg/Al nonchromatic X-ray source, and an Omicron EA 125 hemispherical electron energy analyzer with a five-channel detector. The XPS spectra were collected in a constant pass energy mode with a pass energy of 30 eV and were calibrated to adventitious C 1s peak at 284.6 eV. All peaks were fit (using CasaXPS software^16^) to symmetric Voigt functions (70% Gaussian and 30% Lorentzian) and a Shirley background to determine peak positions and areas. The fitting parameters were generated with a Levenberg–Marquardt optimization algorithm.

### 2.4 Electrical Resistivity Characterization

To characterize the electrical resistances of SBO NPs, electrochemical impedance spectroscopy (EIS) measurements were performed on a lithium coin cell battery using a BioLogic SP-150 potentiostat/galvanostat with an oscillation voltage of 10 mV and a frequency range from 10^5^ to 10^–2^ Hz. All electrochemical measurements were carried out at room temperature and held at the open-circuit voltage for 30 min. The working electrodes were prepared by mixing active materials, carbon black, and poly(vinylidene fluoride) in a weight ratio of 70:20:10 in 400 μL of 1-methyl-2-pyrrolidinone (NMP, Sigma-Aldrich, anhydrous, 99.5%) to form a slurry. The mixed slurry was coated uniformly onto a thin copper foil, dried overnight in the air at 100 ^o^C. Coin cell batteries (CR2032/CR2016) were assembled using a working electrode, a polypropylene microporous film as the separator, and a lithium foil as the counter and reference electrode. A 1 M solution of LiPF_6_ dissolved in ethylene carbonate and dimethyl carbonate (1:1 in volume ratio) was used as the electrolyte. The Li-ion half-cells were assembled in an Ar-filled glovebox with both water and oxygen contents below 40 ppm. The collected spectra were simulated and fit with an equivalent circuit using the ZView software (Scribner Associates Inc.). The electrical resistivity was further measured using a Quantum Design Physical Property Measurement System (QD PPMS) and a KEITHLEY 2601A multimeter. The powder samples were uniaxially pressed into pellets (∼100 mg, Ø ∼6.5 mm × 0.9 mm) at 1 ton and transferred to a quartz tube furnace to be annealed at 800 °C for 8 h in N_2_ atmosphere.

### 2.5 Electronic Structure and Transport Calculations

Planewave DFT calculations were performed using the Vienna Ab-Initio Simulation Package (VASP)^17,18^ to calculate the electronic structure of SBO NPs. These calculations used the Perdew–Burke–Ernzerhof (PBE) functional^19^ to account for exchange and correlation and the projector augmented wave (PAW) method^20^ to describe wave functions in atomic core regions. The Na 3s2p, Sr 4s4p5s, Nb 4p5s4d, Mo 4p5s4d, Ta 6s5d, and O 2s2p orbitals were treated as valence states. All calculations were performed with a planewave cutoff energy of 396 eV and a 3 × 3 × 3 Γ-centered *k*-point grid. A 2 × 2 × 2 supercell, with experimentally determined unit cell lattice parameters,^1,21−23^ was utilized in these calculations. For the determination of the Kohn–Sham orbital populations, a Methfessel–Paxton (second-order) method^24^ was used with a smearing width of 0.2 eV. The atomic positions in the supercell were optimized until the force on each atom was less than 0.05 eV/Å. The high-symmetry *k*-points in the Brillouin zone for the calculated band structure were generated using the automatic-flow for material discovery (AFLOW) software.^25^ Electronic transport properties were subsequently calculated using the semiclassical Boltzmann transport theory within the rigid band and constant relaxation time approach as implemented in the BoltzTraP2 code.^26,27^ A constant relaxation time (τ) of ∼4 fs was used for all calculations, which is suitable for these SBO perovskites.^28^

## 3 Results and Discussion

**Figure 1:**
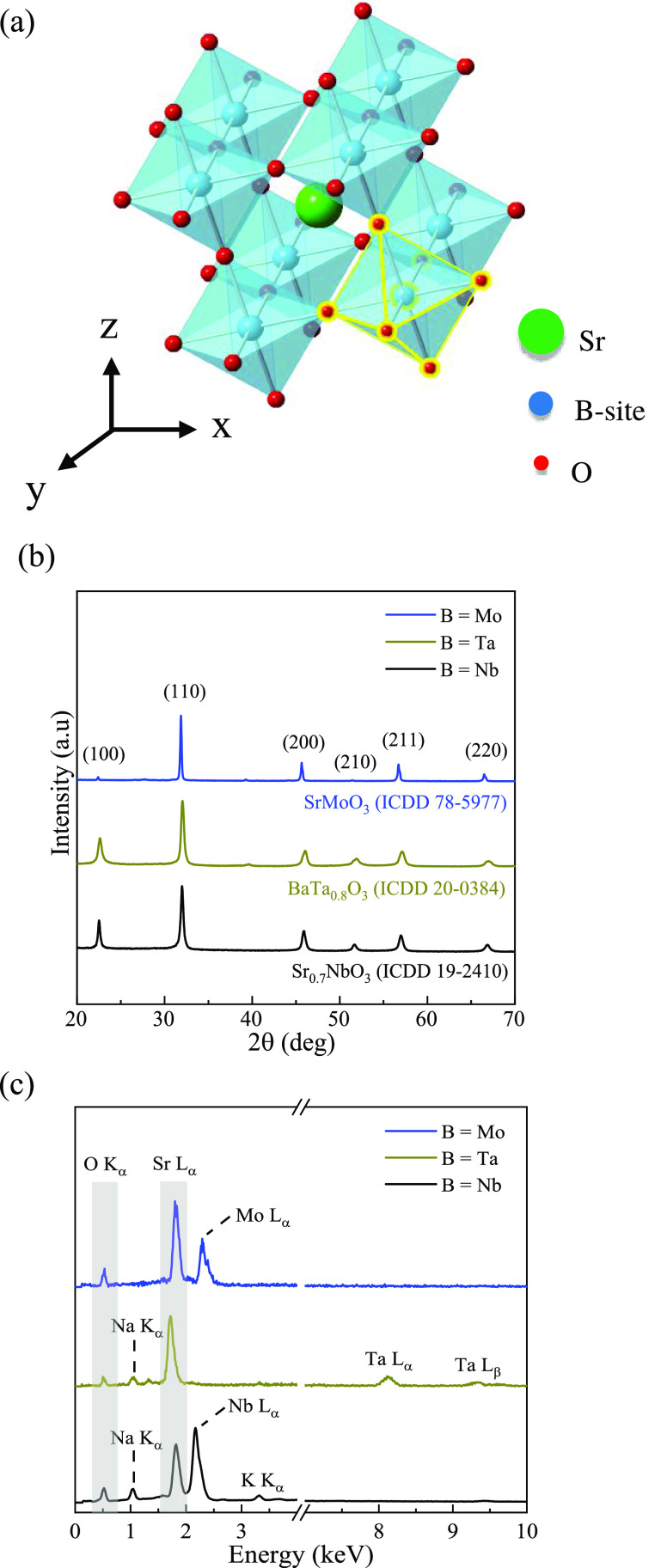
(a) Crystal structure of metastable SBO perovskite and (b, c) XRD pattern and EDX for SBO (B = Nb, Ta, and Mo) NPs synthesized using the two-step coprecipitation/molten salt method followed by a H_2_/Ar post-treatment.

SBO NPs (B = Nb, Ta, and Mo) were synthesized using a two-step coprecipitation/oxygen-controlled molten salt technique^7^ followed by a H_2_/Ar post-treatment (800 °C for *t* = 6 h, Figure 1a). As shown in Figure 1b, the prepared samples possess reflections that match with reported crystallographic references (B = Nb, ICDD 19-2410; B = Ta, ICDD 20-0384; B = Mo, ICDD 78-5977), which is subsequently verified using a combination of Rietveld refinement and ICP-OES analyses. Based on the statistical and visual agreements (Tables S1–S3), the stoichiometries for NPs were determined to be cubic *Pm-3m* Sr_0.7_NbO_3_ (B = Nb, *a* = 3.955(4) Å), Na_0.9_Sr_0.1_(Na_0.4_Ta_0.6_)O_3_ (B = Ta, *a* = 3.946(4) Å), and SrMoO_3_ (B = Mo, *a* = 3.976(0) Å). TEM (Figure S2) and EDX (Figure 1c) results show that the synthesized NPs have particle sizes of ∼20 nm with the expected elements present. The observed Na and K fluorescence in the B = Nb sample is attributed to residual salts from the molten salt bath instead of the incorporation of both cations into the crystal.

**Figure 2:**
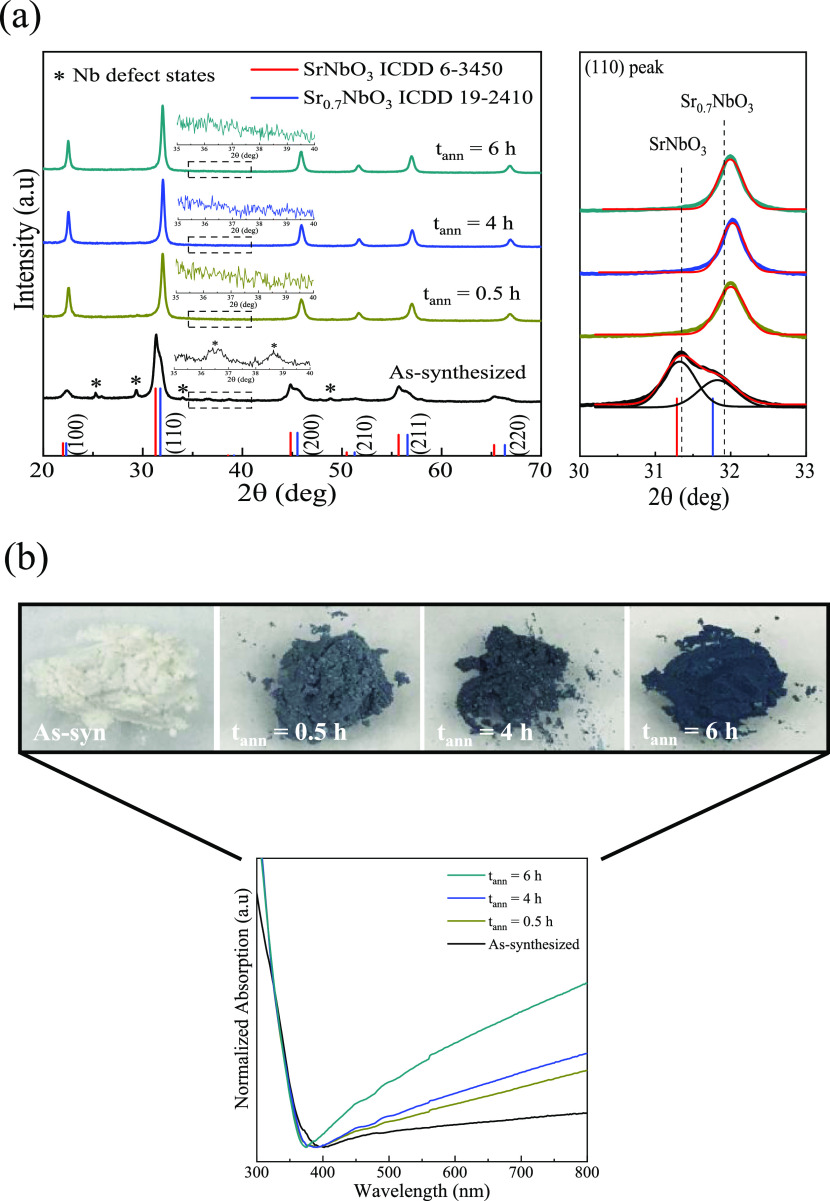
(a) XRD patterns and (b) optical absorption spectra for as-synthesized and H_2_/Ar treated (*T* = 800 °C, *t*_ann_ = 0.5, 4, and 6 h) SNO NPs. The dashed lines are a guide to the eye for bi-phase Sr_0.7_NbO_3_–SrNbO_3_ to single-phase Sr_0.7_NbO_3_ transition. The corresponding powder color is shown in the absorption plot, and the annealing time is displayed at the bottom left corner of each image.

To systematically study the effect of the H_2_/Ar treatment time on the structural (surface and deep defect states) and optoelectronic (insulating and metallic-like states) properties of SBO NPs, the B = Nb (Sr_0.7_NbO_3−δ′_, SNO) sample was reduced at two intermediate times, *t* = 0.5 and 4 h. The crystal structures (Figure 2a), refined using SrNbO_3_ and Sr_0.7_NbO_3_ as structural models (Figure S3 and Table S4), show a shift to the single-phase Sr_0.7_NbO_3_ after a 0.5 h reduction. The formation of the Sr_0.7_NbO_3_ crystal phase after 0.5 h suggests that Nb defect states, likely present at the surface, can be readily reduced. Quantification of the reduction process (Figure S4) shows a persistent weight loss (∼1.2%) between 250 and 800 °C, which is attributed to the partial removal of oxygen from the SNO lattice as a result of reduction to the multivalent B-site cation,^29^ with weight loss below this range associated with physi-/chemisorbed water. The broad exothermic peak positioned at ∼470 °C is ascribed to the subsequent bonding of mobile Sr and O atoms with surface Nb defects,^30^ thus forming a Sr–Nb–O framework. No phase changes are observed as a result of this loss in lattice oxygen ions, which is seen during ion exsolution.^29,31−33^ The loss in lattice oxygen ions persists at 800 °C for ∼5 h, as demonstrated by the additional weight loss of ∼1.2% (corresponding to an overall oxygen deficiency,^33^ δ′ ∼ 0.34). The observed δ′ also agrees with iodometric titration analysis of SNO NPs treated under similar conditions (Table S5). While no obvious weight loss was detected with further increase to the reduction time (>5 h), suggesting completion of the reduction process,^29^ the observed increased heat flow during this time is most likely due to continued formation of the Sr–Nb–O network from deep Nb defects. This trend is consistent with B = Mo (SMO) and Ta (STaO) perovskites, highlighting that it is not a function of the B-site ion.

In addition to reducing multivalent Nb sites, a systematic change in the powder color (i.e., white to blue) and optical absorption occurred (Figure 2b). These reduced SNO NPs have a similar spectrum in the UV region (300–400 nm) but exhibit significant enhancement in their optical absorption in the visible to a near-infrared region (400–800 nm) as a function of the annealing time. These changes in the absorption profile coincide well with experimental and theoretical reports.^1,2,34−36^ The first noticeable color change (from white to light blue, *t* < 0.5 h) is ascribed to the removal of surface defects that are periodically arranged within the lattice (Figure 2a). These defect sites are eliminated during the H_2_/Ar treatment, transforming the initially white powder (insulating) to a light blue (conductive). With *t* > 0.5 h, the powder becomes darker, which is attributed to the subsequent partial removal of deep defects and reordering of the bulk crystal. Furthermore, the STaO (white to gray) and SMO (white to black) samples exhibited similar responses to SNO (Figure S5). The observed color changes did not fade even after exposing the powders to air for more than 6 months, suggesting that the crystals are highly stable under ambient conditions.^37^ However, after annealing metastable crystals in air, they revert back to their thermodynamically stable (white powder) state, similar to previous reports, which is likely due to oxidation of B-site cations.^1,2,38^ From the above analysis, the observed optical changes are clear indicators of a gradual structural transformation induced from the suppression of defect states (surface, *t* < 0.5 h and deep, *t* > 0.5 h).

**Figure 3:**
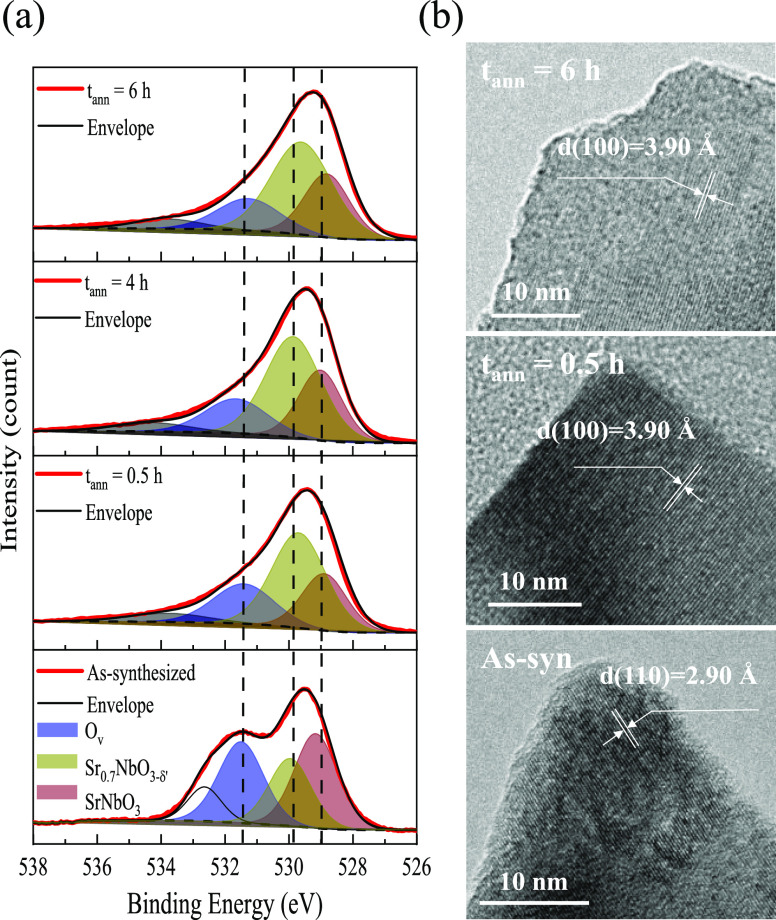
(a) XPS O 1s spectra and (b) HRTEM images for as-synthesized and H_2_/Ar-treated SNO NPs. The dashed lines in the XPS spectra are a guide for the eyes. The HRTEM images with short (0.5 h) and long (6 h) H_2_/Ar annealing times highlight the absence of exsolved metallic particles and only show a homogeneous Sr_0.7_NbO_3−δ′_/SrNbO_3_ crystal.

Previous studies have demonstrated that oxygen vacancies (V_O_) can be generated in metal oxides under oxygen-reduced conditions resulting in changes to the electronic structure and powder appearance.^5,9,12,31,37^ Therefore, to verify that visual changes are not due to the formation of V_O_, the binding environment of O in SNO NPs was probed (Figure 3a). The deconvoluted O 1s XPS spectra of as-synthesized NPs identifies four peaks assigned to SrNbO_3_ lattice oxygen (529.2 eV), Sr_0.7_NbO_3−δ′_ lattice oxygen (530.0 eV), oxygen defects (531.5 eV), and surface hydroxyls (532.6 eV).^7,39^ The surface hydroxyl peak is removed during the reduction treatment with the remaining peaks unchanged. The removal of the hydroxyl peak after 0.5 h corresponds with the initial change in the visual appearance suggesting that surface defects significantly impact electronic properties. The changes in the area ratio of main SNO lattice oxygens (Sr_0.7_NbO_3−δ′_ and SrNbO_3_) for the as-synthesized compared with the treated samples are consistent with the crystal structure evolution (Figure 2a). The V_O_ peak area sharply decreases upon reduction (0.5 h, −36%) with relatively little change throughout the remaining reduction process (Figures 3a and S6). The fact that the V_O_ peak area does not increase during the reduction processes indicates that the formation of oxygen defects does not contribute to the optical response. Additionally, HRTEM images of reduced SNO NPs show *d*-spacings of 2.90 and 3.90 Å corresponding to the (110) and (100) lattice planes of Sr_0.7_NbO_3−δ′_ (Figure 3b). The homogeneous crystal composition observed in the images further confirms the absence of exsolution-induced phase changes as a result of the extended treatment.^29,31,32^ These results reveal that although V_O_-type defects are present, the optical changes from the reduction process are a result of suppressing surface/deep defect states. The proposed reduction mechanism for these SNO NPs is summarized in Figure S7. Upon heating as-synthesized NPs in an oxygen-reduced environment, Sr and O atoms diffuse across the grain boundaries, presumably from the stoichiometric to the Sr-deficient phase. Then, the diffused atoms bind with surface Nb defects at ∼470 °C, forming a stable Sr–Nb–O network (Figure S7a). The fact that this process is relatively fast (*t* < 0.5 h) suggests that the loss of chemisorbed hydroxyl groups occurring at this temperature makes the bond formation step kinetically favorable. This bond formation induces structural transformations that drive the appearance of the metallic character (color change).^40^ With increased treatment time, the network expands by binding the diffused atoms with defects located deeper within the crystal. This process is kinetically slow based on the longer times needed (*t* > 0.5 h). In the final step, the bi-phasic structure is reorganized into a single-phase Sr-deficient lattice (Sr_0.7_NbO_3−δ′_, δ′ ∼ 0.34; Figure S7b) with no other observed crystal changes.^31−33^ The δ′ agrees with the previously reported oxygen deficiency (δ ∼ 0.35),^7^ further demonstrating that the Sr-deficient lattice is the final crystal structure. Thus, the structural and optical results provide strong evidence that these SBO NPs potentially undergo an IMT (white, insulating to colored, metallic-like) when thermally treated in an oxygen-reduced environment.

**Figure 4:**
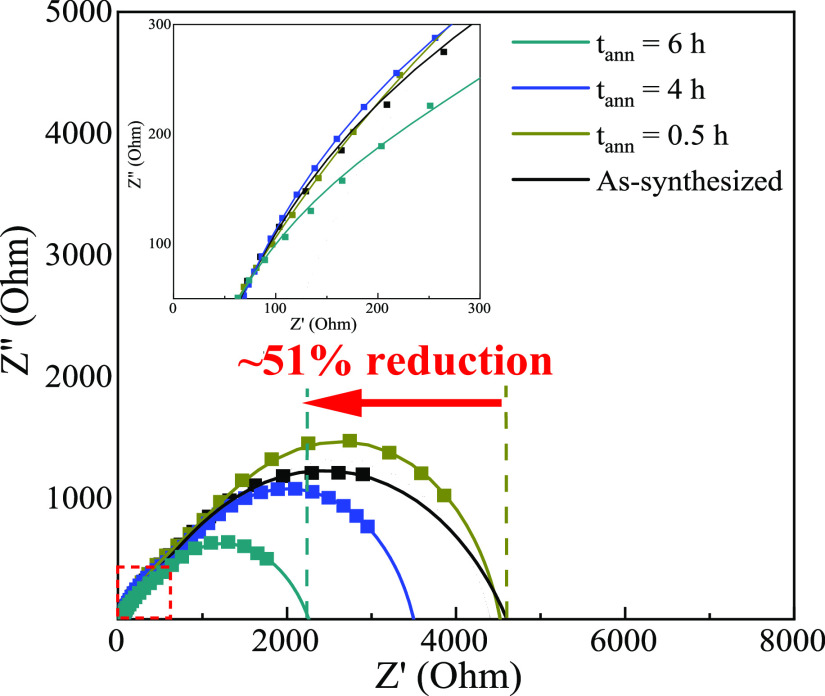
EIS curve for as-synthesized and H_2_/Ar-treated SBO (B = Nb). The inset presents an enlarged view of the high-frequency region of the EIS spectra (red dashed box).

EIS spectroscopy was performed to further characterize the electrical activity during the IMT for these NPs. This technique has been used to monitor changes in the electronic conductivity of reduced W_18_O_49_,^5^ TiO_2_,^37^ and SrTiO_3_.^41^ The corresponding Nyquist plots for the reduced SNO NPs are shown in Figure 4. The cell configuration and equivalent circuit model employed to characterize the Li-ion half-cell are presented in Figure S8.^42^ The model incorporates the resistances for the electrolyte (*R*_S_), solid-electrolyte interface (SEI, *R*_SEI_), anode/cathode charge transfer (*R*_CT_), and constant phase elements (CPE_SEI_ and CPE_ct_). The electrical resistances extracted from the optimized fit (χ^2^ < 10^–3^) are summarized in Table S6. The EIS measurements focused on the high-frequency region (>200 mHz) where the electrolyte, SEI, and anode/cathode electrode^43^ are dominant and excluded the low-frequency Li-ion diffusion.^37^ The slight increase in capacitance observed after the 0.5 h treatment is attributed to the reduction of surface defects, which hinders bulk charge transport.^44,45^ This capacitive behavior subsequently decreases proportionally with annealing time due to the suppression of bulk defects, facilitating the depletion of accumulated surface charges.^46^ After the 6 h treatment, a homogeneous structure is obtained, which can effectively transport charges and reduces the total capacitance. As expected, the enhanced electronic conductivity of NPs also contributes to systematically reducing the electrode cell resistance. Specifically, the *R*_CT_ of the as-synthesized SNO electrode (4.31 kΩ) is reduced to 254 Ω after 6 h, demonstrating that the suppression of deep defects substantially reduces charge transfer resistances. Conversely, an increase (from 252 Ω to 1.97 kΩ) in the *R*_SEI_ is observed as a result of the IMT, which is associated with enhanced electrical transport that leads to considerable electrolyte decomposition and SEI growth.^47^ As seen from changes in the capacitance and resistance (*R*_CT_, *R*_SEI_), when *t* < 0.5 h, the electrical activity of NPs is driven by surface transport. Unfortunately, surface defects act as trap states that localize free carriers,^9^ thus reducing carrier mobility and impeding bulk transport. However, when *t* > 0.5 h, bulk transport dominates and a dramatic change in electronic properties, i.e., an insulating to metallic-like state, is observed. The suppression of deep defects improves charge transport by increasing the number of free carriers and carrier mobility. Similar to SNO, qualitative and quantitative variations were observed for the reduced STaO and SMO NPs as a result of their modified electronic properties (Figure S9 and Table S7). However, Mo and Ta crystals show reduced *R*_CT_ compare to Nb samples, with the resistance of STaO crystals nearly a quarter of the SNO and significantly lower than literature values for similar systems (Table 1). These results suggest that the IMT is largely influenced from the inhibition of bulk rather than surface defect formation.

**Table 1:** Calculated and Measured Electronic Properties for SBO NPs

crystal structures	measured *R*_CT_ (Ω)	reported *R*_CT_ for metal oxide anode materials (Ω)^a^	measured ρ for SBO (Ω cm)	calculated ρ for SBO (Ω cm)^b^	reported ρ (Ω cm)	refs
B = Nb	254.2	binary: Ti/V/Mo/Sn–O systems (∼300–1400)	1.1 × 10^3^	(1.7–2.1) × 10^–4^	(∼1.0–69.0) × 10^–4^	(48−52)
B = Mo	173.8	tertiary: H–Ti–O systems (∼300–1400), Li–Ti–O systems (∼107–219), Ti–Nb–O systems (∼150–700)	1.7 × 10^–4^	1.7 × 10^–4^	(∼0.2–2.0) × 10	
B = Ta	68.1		c	2.1 × 10^–2^–1.5 × 10^–4^	(∼12) × 10^–4^	(50, 53−58)

a Reported *R*_CT_ values using the same Li-ion battery assembly as in the current study.

b The two resistances shown for STaO are representative of the defect-free (1.5 × 10^–4^) and defective (2.1 × 10^–2^) crystals.

c Nonconstant ρ (i.e., resistance varies with voltage).

**Figure 5:**
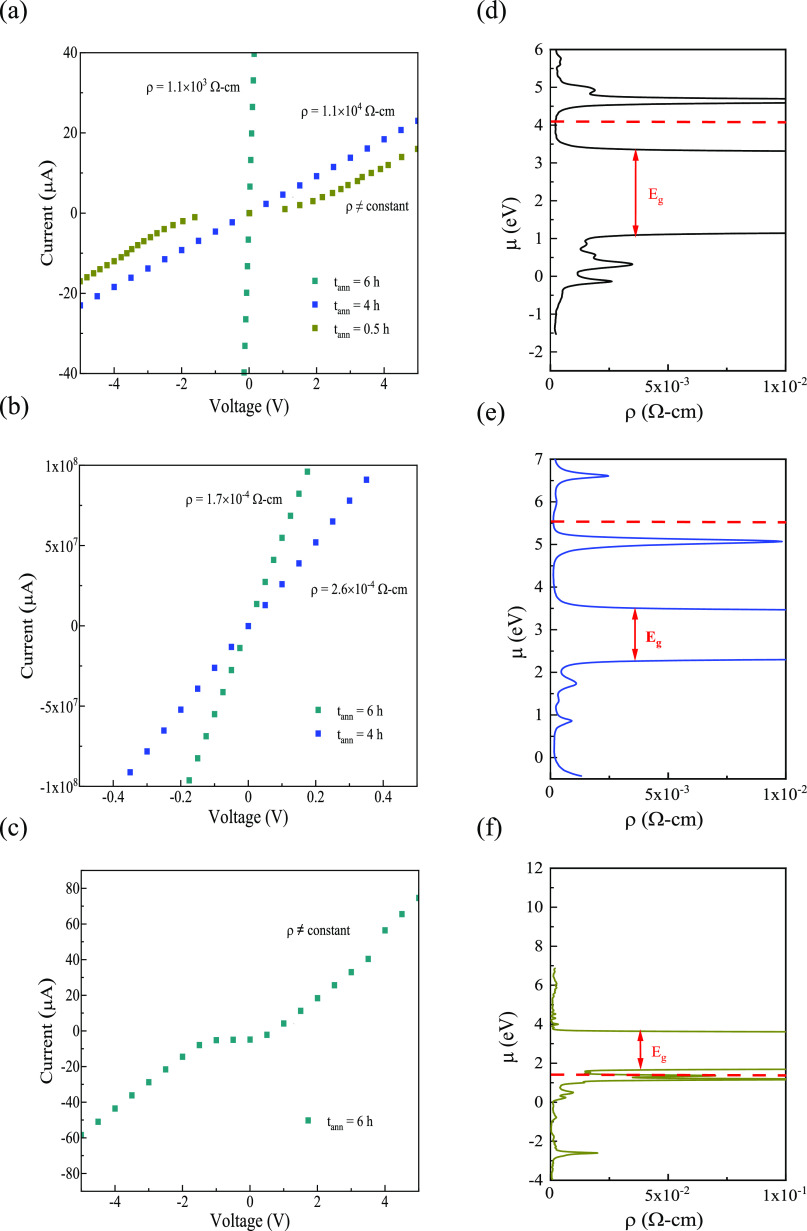
Measured and calculated electrical resistivity (ρ) for defective SBO NPs: (a, d) B = Nb, (b, e) B = Mo, and (c, f) B = Ta. The ρ calculations were performed at 300 K. The Fermi level is indicated with a dashed red line in the plots.

The induced metallic-like nature of these SBO NPs was further investigated by measuring the current–voltage (*I*–*V*) response. The electrical resistivities (ρ), plotted from −5.0 to 5.0 V, were calculated by applying Ohm’s and Pouillet’s laws.^59^ The current value for as-synthesized SBO NPs was beyond the detection resolution of the instrument due to their intensely insulating nature, but demonstrated a systematic decrease in resistivity (1.1 × 10^3^ Ω cm for 6 h) with treatment time (Figure 5a). Although a similar quantitative ρ reduction was observed for SMO (from 2.6 × 10^–4^ Ω cm for 4 h to 1.7 × 10^–4^ Ω cm for 6 h; Figures 5b and S10), a nonohmic response was observed for the STaO after 6 h (Figure 5c). It is important to note that the measured ρ for SMO is considerably lower than the ρ for SNO and STaO. The measured ρ for SMO is in good agreement with the thin-film counterpart,^21^ signifying the complete removal of deep defect states in the sample. However, in the case of SNO and STaO, the ρ is higher than what has been reported in literature.^1,22^ From these results, it is evident that the defect stability in these metastable crystals follows the order of Mo < Nb < Ta. This trend is closely correlated with the descending sequence of electronegativity observed for the B-site ions (Mo ∼ 2.2; Nb ∼ 1.6; Ta ∼ 1.5).^60,61^ Therefore, as the electronegativity of the B–O bond increases, it presumably becomes more challenging to stabilize defects during the H_2_/Ar treatment, thus facilitating the crystal formation.^8,62^ However, the identity of these deep defect states, and their impact on the electronic structure, is not immediately clear based on these experiments.

To understand how the structure and defects are impacting the electronic properties, electrical resistivities (ρ) were extracted from DFT calculations. No Hubbard *U* correction^63^ was included in these calculations,^36^ starting with stoichiometric SBO (Figure S11a–c). Comparison of these results (calculated optical transition: SNO, *E*_g_ ∼ 2.3 eV; STaO, *E*_g_ ∼ 3.0 eV; SMO, *E*_g_ ∼ 1.1 eV) with computational reports^38,64,65^ show good agreement. Although *E*_g_, which arises from the O 2p–B 4d or 5d t_2g_ interband transition, is experimentally observed to be >3–4 eV,^65^ DFT is known to underestimate this transition energy. Furthermore, ρ values were calculated by utilizing the Boltzmann transport theory as implemented in the BoltzTraP2 code (Figure S11d–f). As displayed in Table 1, the calculated ρ at the Fermi level is consistent with reported values.^36,65,66^ Then, to mirror the measured SBO stoichiometries (Tables S3 and S5), the following models were utilized: (i) for SMO, no Sr (V_Sr_) and O (V_O_) vacancies were incorporated into the corresponding defect-free model, (ii) for SNO, 2 V_Sr_ and 2 V_O_ vacancies were introduced into the corresponding defect-free model, and (iii) for STaO, 10 Na atoms (7 A-site, 3 B-site) and 5 V_O_ were introduced into the corresponding defect-free model. The calculated band structures and density of states (DOS) for defective SBO NPs show that the Fermi level sits in the respective bands (Figure S12a–c, dashed red line), revealing that the observed electronic conductivity of these metastable crystals is a result of n-type (SNO and SMO) and p-type (STaO) behavior. Additionally, in comparison to reduced STO and TiO_2_,^10,13^ the Fermi level for these defective SBO perovskites lies deep within either the conduction or the valence band. For SNO and SMO, the n-type conductivity arises from the partially occupied t_2g_ bands of the metal d orbitals.^21,66^ In the case of STaO, it is believed that the partial substitution of Na into the Sr/Ta-sites of STaO significantly dopes hole carriers into the top of the valence band, resulting in the p-type conductivity.^67^ From the above calculations, the electronic structure of metastable SBO perovskites is determined to be conducive to n- or p-type transport, depending on the nature of the B-site ion, making them robust conductors.

Similarly, the ρ for defective SBO perovskites were calculated and presented in Figure 5d–f and Table 1. According to these transport calculations, the ρ of STaO is higher than that of SNO and SMO, which is consistent with the response observed experimentally. The calculated ρ for the SMO agrees with the measured values, further highlighting the absence of deep defect states in the sample after the H_2_/Ar treatment. Thus, when these defect states are fully suppressed, the two electrons per B^4+^ ion that Mo possesses can contribute to significantly increasing its free carrier density.^66^ However, in the case of SNO and STaO, the deep defect states are more difficult to fully suppress, resulting in the observed difference between the experimental and calculated ρ values. This behavior is consistent with other SNO calculations that reported inhibited electronic transport due to the presence of bulk defect states.^1^ These results demonstrate that the variation in the measured electrical resistivity of SBO NPs can be explained by changes in deep defect states. Thus, the computational findings coincide well with the experimental observations noted above.

Overall, these results effectively demonstrate the ability to synthesize metastable ABO_3_ perovskites and systematically modify their electronic properties, which have potential applications in future electrochemical devices. As an example, the modified electronic properties of these SBO NPs result in transport properties, which offer lower energy barriers for Li-ion insertion/extraction (i.e., reduced *R*_CT_),^68^ making them favorable anode materials for Li-ion batteries. Moreover, charge transport properties show significant improvement over reported metal oxide anode materials (Table 1) due to the ability to control defect concentrations which can be used to tune the p-/n-nature of the materials.

## 4 Conclusions

These conductive metal oxides are of interest because they are technologically significant for next-generation electrochemical devices. While metallic properties are observed in thin-film/bulk analogues of these SBO perovskites, NPs synthesized using traditional wet-chemical methods appear to be insulating, resulting from B-site atom oxidation states. These results demonstrate that under oxygen-reduced conditions (H_2_/Ar), an IMT can be induced in as-synthesized SBO NPs, which is driven by the suppression of surface/deep defect states. Specifically, surface defect states, which are partially stabilized by hydroxyl groups, can be quickly removed using a H_2_/Ar post-treatment (treatment time, *t* < 0.5 h). This rapid treatment results in a noticeable change in the powder color. Based on the optical absorption and electrical activity of NPs, deep rather than surface defect states must be suppressed to induce metallic-like characteristics, necessitating the use of *t* > 0.5 h. As a result of the H_2_/Ar atmosphere, no exsolution-induced phase changes occurred further pointing to an IMT. DFT calculations reveal that n-type (B = Nb and Mo) and p-type (B = Ta) conductions are responsible for the observed metallic-like behavior. Finally, DFT and Boltzmann transport calculations further demonstrate that the expected electronic transport properties of NPs are quenched due to overoxidized B-site cations present in the bulk of the crystal. These findings provide a new approach for the development of novel electrochemical materials via the control of IMT in ABO_3_ perovskites.

## Supporting Information Available

Rietveld refinement and crystallographic/structural parameters for SBO (B = Nb, Ta, and Mo) from GSAS II, corresponding TEM images, TGA–DSC curve, ICP-OES and iodometric titration results, UV–vis spectra for SMO and STaO, schematic representation of the Li-ion battery, EIS equivalent circuit, electrode resistances for SMO and STaO, full IV curve for SMO, schematic representation for proposed mechanism, and electronic structure and transport calculation for SBO supercells (PDF)
