# Supplementary material for: Modifying Metastable Sr1–xBO3−δ (B = Nb, Ta, and Mo) Perovskites for Electrode Materials
Source: ACS Appl Mater Interfaces. 2021 Jun 16;13(25):29788–97. doi: 10.1021/acsami.1c05743 (PMC8289236; doi:10.1021/acsami.1c05743)
Supplement: Supplementary file 1 — am1c05743_si_001.pdf [file am1c05743_si_001.pdf]

Supporting Information for

Modifying Metastable $Sr_{1-x}BO_{3-\delta}$ (B=Nb, Ta, and Mo) Perovskites for Electrode Materials

Tochukwu Ofoegbuna,[1] Benjamin Peterson,[1] Natalia da Silva Moura,[1] Roshan Nepal,[2] Orhan Kizilkaya,[3] Carsyn Smith,[4] Rongying Jin,[2] Craig Plaisance,[1] John C. Flake,[1] and James A. Dorman [1,*]

[1] Cain Department of Chemical Engineering, Louisiana State University, Baton Rouge, Louisiana 70803, United States.

[2] Department of Physics and Astronomy, Louisiana State University, Baton Rouge, Louisiana 70803, United States.

[3] Center for Advanced Microstructure Devices, Louisiana State University, Baton Rouge, Louisiana 70803, United States

[4] St. Joseph's Academy, Baton Rouge, Louisiana 70803, United States.

* Corresponding Author

Email: jamesdorman@lsu.edu.

Figures

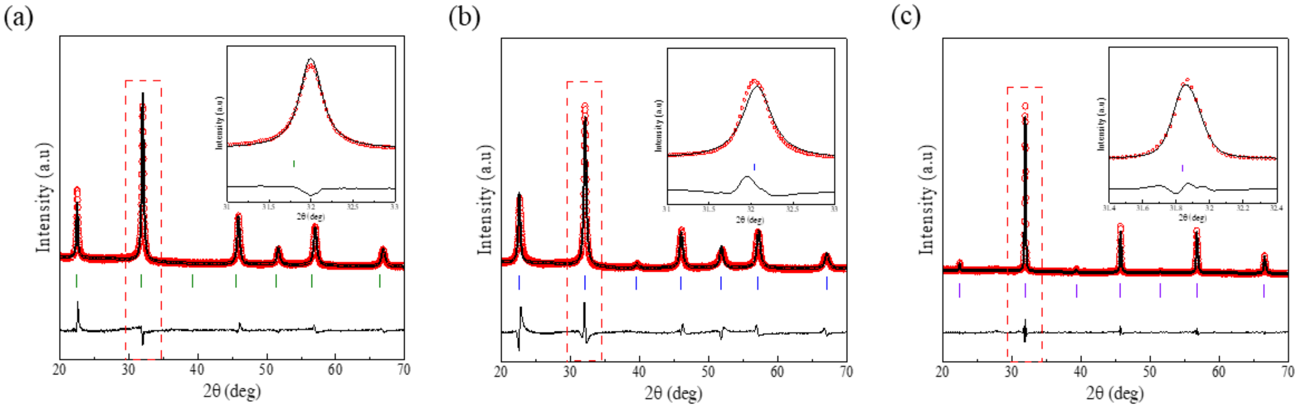

Figure S1. Rietveld refinement for SBO (B = Nb, Ta, and Mo) NPs. The upper symbols illustrate the observed data (circles) and the calculated pattern (solid line). The Bragg reflections ($Sr_{0.7}NbO_3$ - green, $Na_{0.9}Sr_{0.1}(Na_{0.4}Ta_{0.6})O_3$ - blue, and $SrMoO_3$ - purple) and difference curve are shown in the plot. Inset shows enlarged view of the (110) peak (red box).

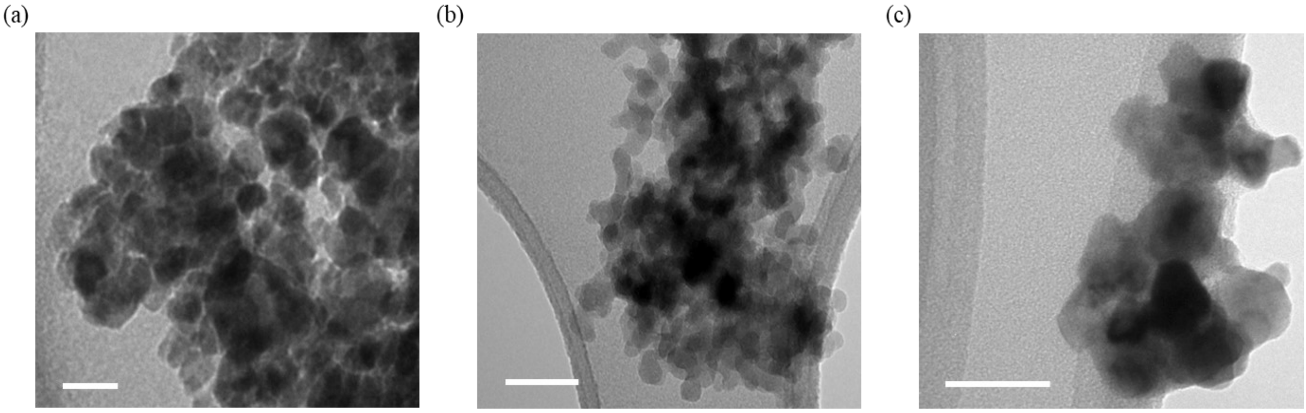

Figure S2. TEM for (a) B = Nb, (b) B = Ta, and (b) B = Mo NPs. The scale bar in the TEM image is 50 nm.

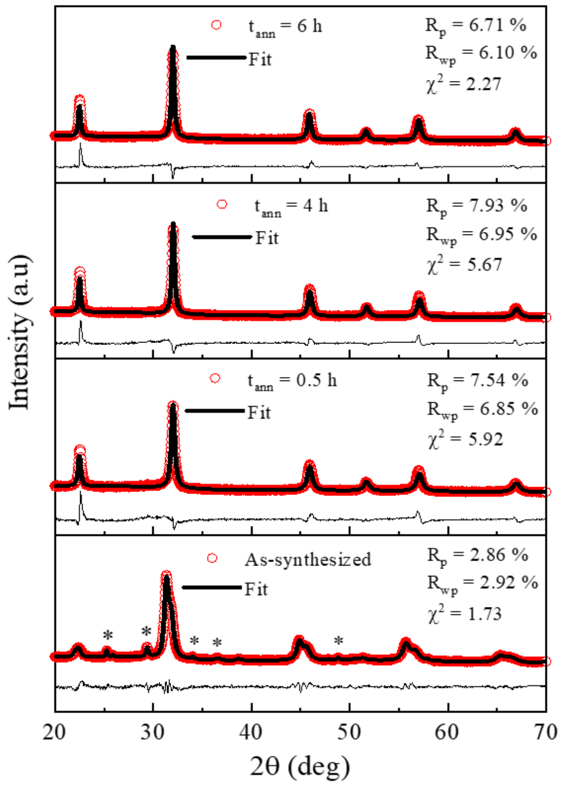

Figure S3. Rietveld refinement for as-synthesized and H_2/Ar treated (T = 800 °C, t_{ann} = 0.5, 4, and 6 h) $Sr_{1-x}NbO_3$ NPs. The upper symbols illustrate the observed data (circles) and the calculated pattern (solid line). The lower curve represents the difference between observed and calculated intensities. Nb^{5+} crystals, present in the as-synthesized samples and removed in the treated samples, are denoted with asterisks.

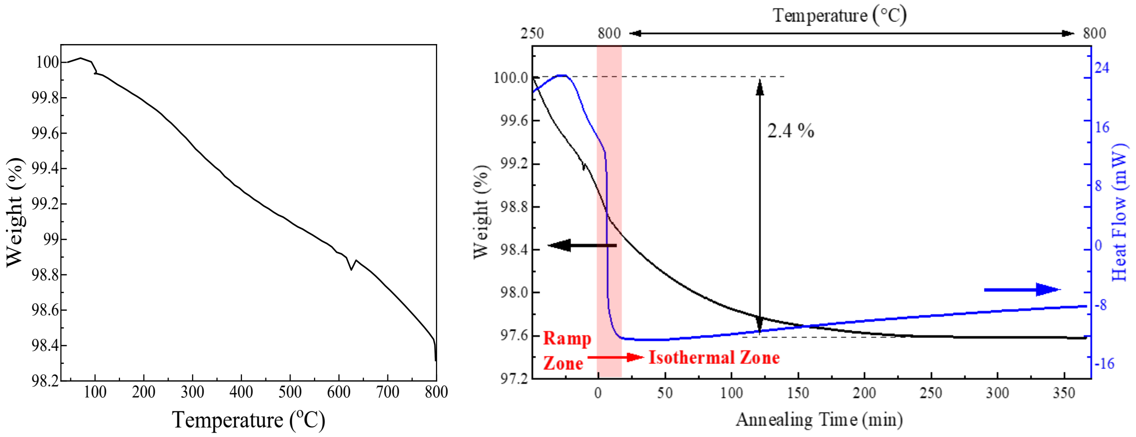

Figure S4. TGA-DSC curve for SNO NPs treated in H_2/Ar atmosphere. (Left) Weight loss is shown as a function of temperature from RT to 800 °C. (Right) Weight loss and heat flow during the full annealing.

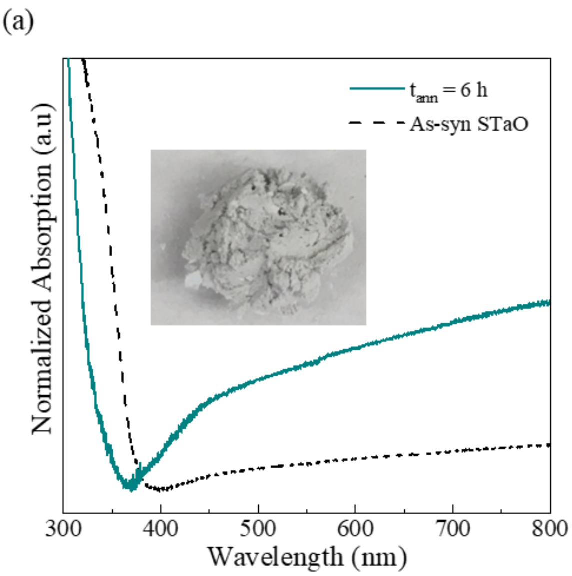

Figure S5. UV-Vis absorption spectrum for (a) B = Ta and (b) B = Mo NPs. The dashed lines in figures a and b are the absorption spectrum for the powders before the H_2/Ar treatment process and the inset shows the treated powder color.

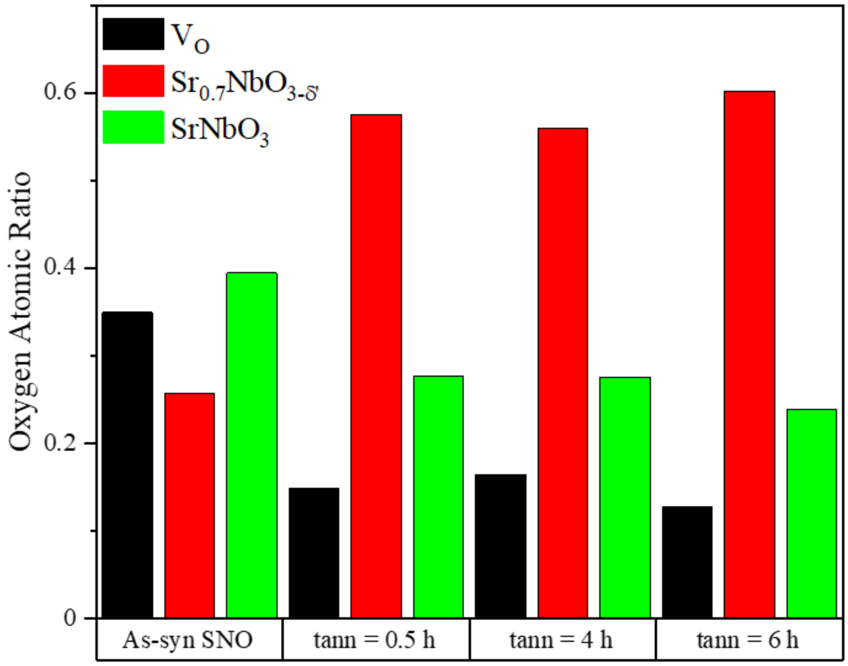

Figure S6. Quantification of atomic ratios for oxygen vacancy and main lattice ($SrNbO_3$ and $Sr_{0.7}NbO_{3-\delta'}$) peaks from deconvoluted O 1*s* XPS data in Figure 3a.

A. Intermediate Structure Formation

$$SrNbO_3 + Sr_{0.7}NbO_{3-\delta} \xrightarrow{H_2/Ar} Sr_{1-x}NbO_{3-\delta'} + Sr - Nb - O \qquad (1a)$$

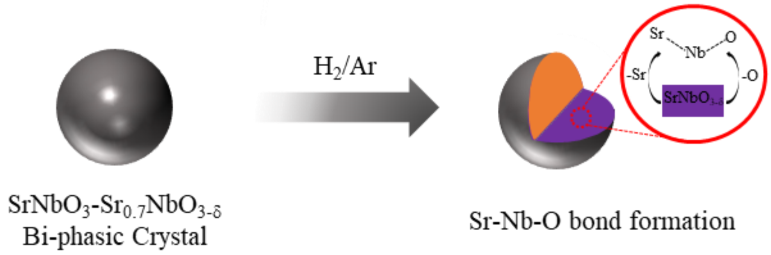

B. Perovskite Lattice Reorganization

$$Sr_{1-x}NbO_{3-\delta'} + Sr - Nb - O \xrightarrow{H_2/Ar} Sr_{0.7}NbO_{3-\delta'} + O_2 \qquad (1b)$$

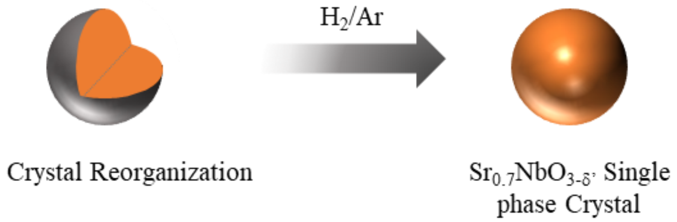

Figure S7. Schematic representation for the proposed mechanism for the treatment of SNO NPs in H_2/Ar atmosphere. The mechanism follows two steps: (a) intermediate structure formation and (b) perovskite lattice reorganization.

(a)

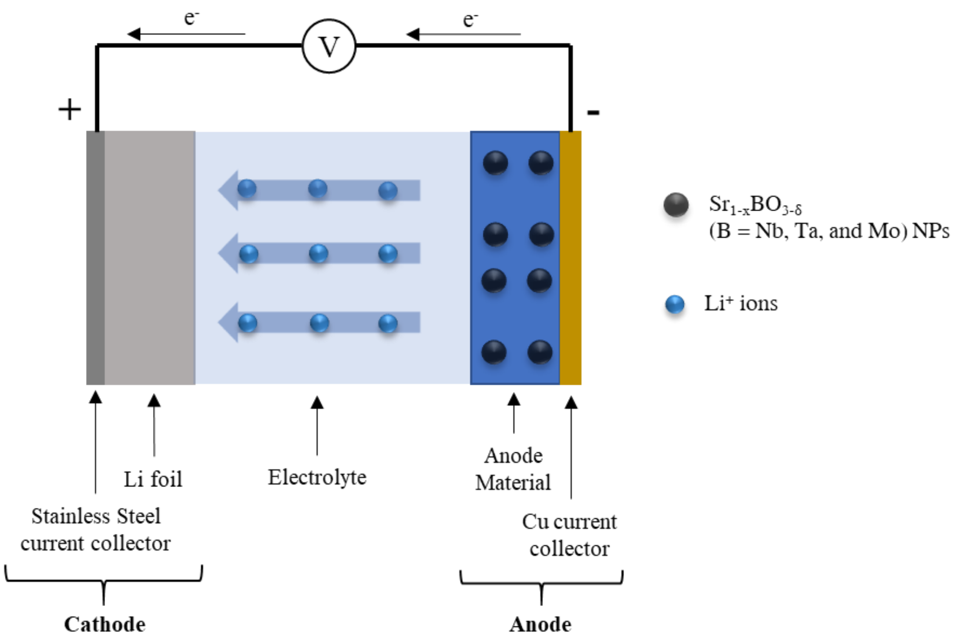

(b)

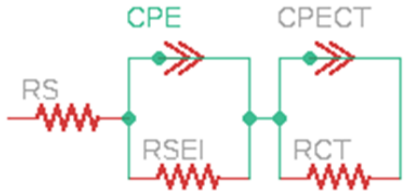

Figure S8. (a) Schematic representation of a Li-ion battery during the discharge process using the SBO (B = Nb, Ta, and Mo) nanoparticles as anode materials and (b) equivalent circuit used to fit the experimental data.

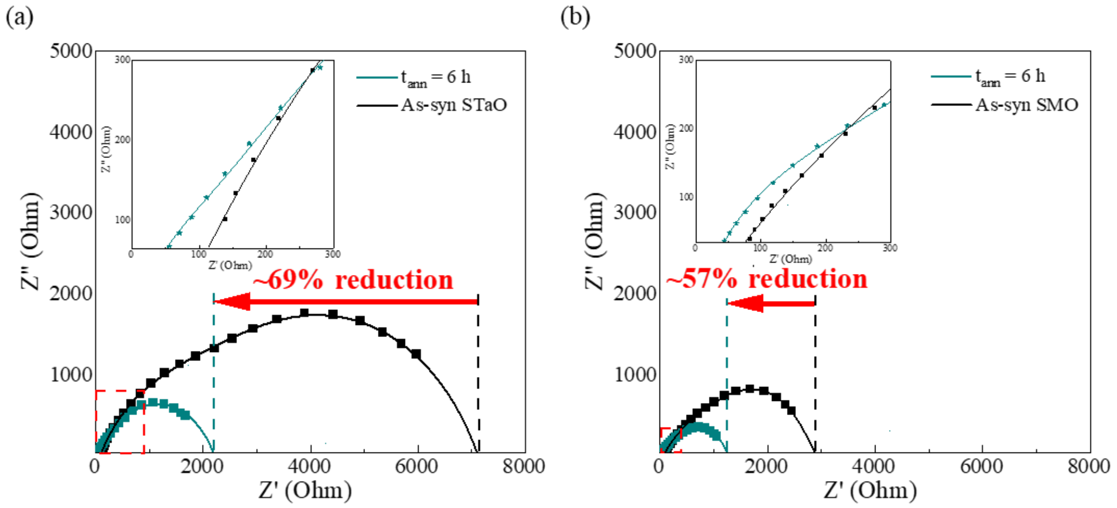

Figure S9. EIS spectra for (a) B = Ta and (b) B = Mo nanoparticles highlighting the IMT. The inset of the EIS curves presents an enlarged view of the high frequency region (red dashed box).

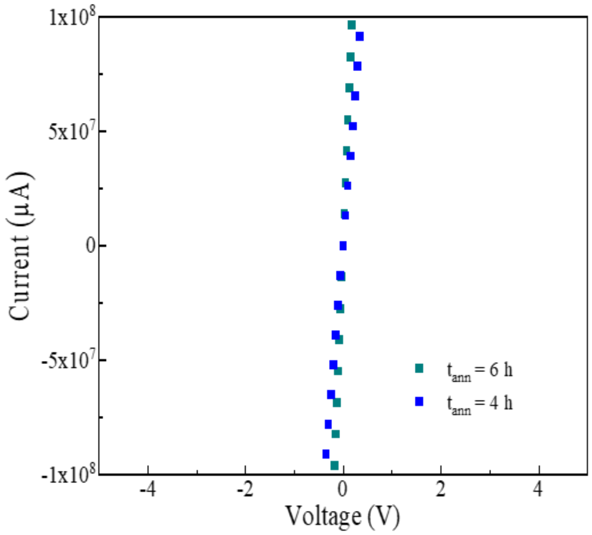

Figure S10. I-V curve for the SMO NPs in the range of -5 to 5 V.

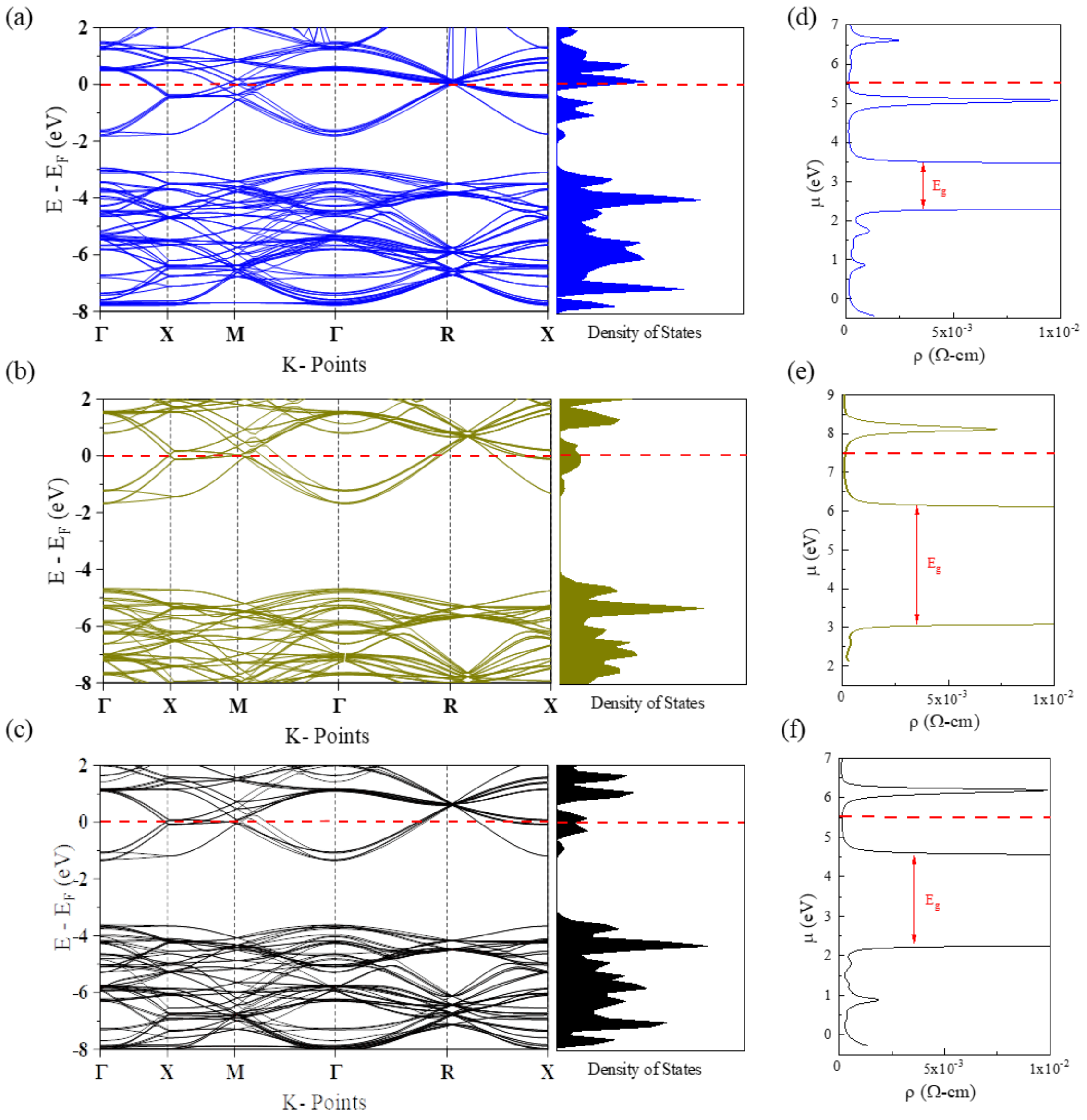

Figure S11. Band structure, total density of state, and electrical resistivity (ρ) calculations for the defect-free SBO NPs: (a, d) B = Mo, (b, e) B = Ta, and (c, f) B = Nb. The ρ calculations were performed at 300 K. The Fermi level is indicated with a dashed red line in all plots.

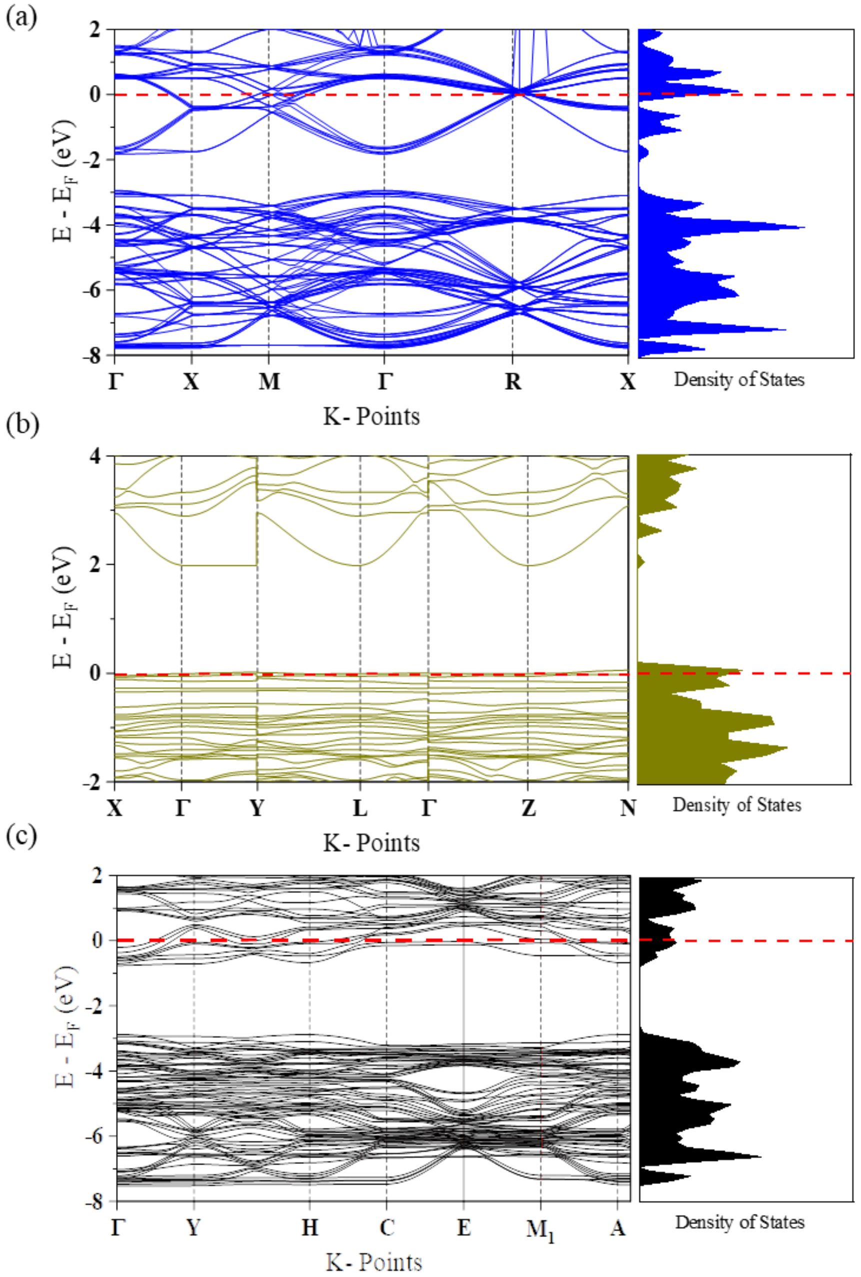

Figure S12. Band structure, total density of states for the defective SBO NPs: (a) B = Mo, (b) B = Ta, and (c) B = Nb. The Fermi level is indicated with a dashed red line in all plots.

Tables

Table S1. Crystallographic Data and Refined Lattice Parameters for SBO (B = Nb and Mo) NPs annealed under H_2/Ar atmosphere based on X-ray Diffraction Data

Parameters	B = Nb	B = Mo
wavelength (Å)	1.541	
temperature (K)	300	
2θ range (˚)	20-70	
space group	Pm-3m (No. 221)	
Z	1.0	
R_p, R_{wp} (%)	6.71, 6.10	5.85, 5.81
χ^2	2.27	1.96
Lattice Parameters		
a, b, c (Å)	3.955(4)	3.976(0)
α, β, γ (˚)	90	90
V (Å^3)	61.881	62.850
Atomic Coordinates		
Sr (x,y,z)	1/2, 1/2, 1/2	1/2, 1/2, 1/2
B (x,y,z)	0, 0, 0	0, 0, 0
O (x,y,z)	1/2, 0 , 0	1/2, 0 , 0
Structural Parameters		
Sr-O (Å)	2.796(9)	2.811(4)
B-O (Å)	1.977(7)	1.987(9)
O-B-O (deg)	180	180
O-B-O (deg)	90	90
B-O-B (deg)	180	180

The occupancy was fixed to 0.7 at the Sr site and 1 at Nb and O sites for the B = Nb structure and 1 at all atom sites for the B = Mo structure. For the B = Nb, the isotropic displacement parameter U_{iso} is 0.071(7), 0.003(3), and 0.029(8) for Sr, Nb, and O sites, respectively. For the B = Mo, the isotropic displacement parameter U_{iso} is 0.016(7), 0.014(8), and 0.025(3) for Sr, Mo, and O sites, respectively.

Table S2. Crystallographic Data and Refined Lattice Parameters for SBO (B = Ta) NPs annealed under H_2/Ar atmosphere based on X-ray Diffraction Data

Parameters	B = Ta
wavelength (Å)	1.541
temperature (K)	300
2θ range (°)	20-70
space group	Pm-3m (No. 221)
Z	1.0
R_p, R_{wp} (%)	11.53, 10.12
χ^2	8.57
Lattice Parameters	
a, b, c (Å)	3.946(4)
α, β, γ (°)	90
V ($Å^3$)	61.463
Atomic Coordinates	
Sr/Na1 (x,y,z)	1/2, 1/2, 1/2
Ta/Na2 (x,y,z)	0, 0, 0
O (x,y,z)	1/2, 0, 0
Structural Parameters	
Sr/Na1-O (Å)	2.791(0)
Ta/Na2-O (Å)	1.973(0)
O-Sr/Na1-O (deg)	90
O-Ta/Na2-O (deg)	90
Sr/Na1-O-Sr/Na1 (deg)	180
Ta/Na2-O-Ta/Na2 (deg)	180

The occupancy was refined for the Sr, Na, Ta, and O sites of the B = Ta structure. The isotropic displacement parameter U_{iso} is 0.094(8), 0.011(8), 0.026(8), 0.012(0), 0.011(4), and 0.024(0) for Sr, Ta, Na1, Na2, and O sites, respectively.

Table S3. ICP-OES Analysis on SBO (B = Nb, Ta, and Mo) NPs annealed under H_2/Ar atmosphere for 6 h

Material	ICP-OES (mg/L)	ICP-OES (mmol/L)	Sr/B Ratio	Measured Stoichiometry
B = Nb	Sr (3.680)<br>Nb (5.776)	Sr (0.0420)<br>Nb (0.0622)	0.7	$Sr_{0.7}NbO_{3-\delta}$
B = Ta	Na (5.260)<br>Sr (0.791)<br>Ta (17.957)	Na (0.2288)<br>Sr (0.0090)<br>Ta (0.0992)	0.1,<br>2.30*	$Na_{0.9}Sr_{0.1}(Na_{0.4}Ta_{0.6})O_{3-\delta}$
B = Mo	Sr (15.074)<br>Mo (18.723)	Sr (0.1720)<br>Mo (0.1951)	0.9	$Sr_{0.9}MoO_{3-\delta}$

*Na was present in high concentrations only in the B = Ta sample, therefore, a Na/Ta ratio was also calculated for this sample.

Table S4. Crystallographic Data and Refined Lattice Parameters for SNO nanoparticles annealed under H_2/Ar atmosphere based on X-ray Diffraction Data

Parameters	t_{ann} = 0.5 h	t_{ann} = 4 h	t_{ann} = 6 h
wavelength (Å)		1.541	
temperature (K)		300	
2θ range (˚)		20-70	
space group		Pm-3m (No. 221)	
Z		1.0	
R_p, R_{wp} (%)	7.54, 6.85	7.93, 6.95	6.71, 6.10
χ^2	5.92	5.67	2.27
	Lattice Parameters		
a, b, c (Å)	3.950(4)	3.950(1)	3.955(4)
α, β, γ (˚)	90	90	90
V (Å^3)	62.850	61.441	61.881
	Atomic Coordinates		
Sr (x,y,z)	1/2, 1/2, 1/2	1/2, 1/2, 1/2	1/2, 1/2, 1/2
B (x,y,z)	0, 0, 0	0, 0, 0	0, 0, 0
O (x,y,z)	1/2, 0 , 0	1/2, 0 , 0	1/2, 0 , 0
	Structural Parameters		
Sr-O (Å)	2.793(4)	2.793(1)	2.796(9)
B-O (Å)	1.975(2)	1.975(0)	1.977(7)
O-B-O (deg)	180	180	180
O-B-O (deg)	90	90	90
B-O-B (deg)	180	180	180

The occupancy was fixed to 0.7 at the Sr site and 1 at Nb and O sites for the t_{ann} = 0.5, 4, and 6 h structure. For the t_{ann} = 0.5 h, the isotropic displacement parameter Uiso is 0.075(4), 0.000(5), and 0.016(8) for Sr, Nb, and O sites, respectively. For the t_{ann} = 4 h, the isotropic displacement parameter Uiso is 0.082(6), 0.002(5), and 0.015(3) for Sr, Nb, and O sites, respectively. For the t_{ann} = 6 h, the isotropic displacement parameter Uiso is 0.071(7), 0.003(3), and 0.029(8) for Sr, Nb, and O sites, respectively.

Table S5. Iodometric Titration on SBO (B = Nb, Ta, and Mo) NPs annealed under H_2/Ar atmosphere for 6 h

**Material**	**Volume $Na_2S_2O_3$ (V, mL)***	**Mole $Na_2S_2O_3$ (CV, mmol)**	**δ**
B = Nb	0.40	0.04	0.36
B = Ta	0.6	0.06	0.74
B = Mo	0	0	0.10

*The volume of the added $Na_2S_2O_3$ was averaged from three trials.

Table S6. Extracted impedance spectroscopy fit parameters for SBO (B = Nb) NPs

**Material**	**R_S (Ω)**	**R_{SEI} (Ω)**	**R_{CT} (Ω)**	**$R_{electrode}$ (Ω)**
As-Syn	41.8	252.1	4314.0	4607.9
t_{ann} = 0.5 h	33.6	1146.0	3346.0	4525.6
t_{ann} = 4 h	48.3	2853.0	600.1	3501.4
t_{ann} = 6 h	38.9	1969.0	254.2	2262.1

Table S7 Extracted impedance spectroscopy fit parameters for SBO (B = Ta and Mo) NPs

Material	R_S (Ω)	R_{SEI} (Ω)	R_{CT} (Ω)	$R_{electrode}$ (Ω)
As-Syn STaO	75.9	1957.0	5094	7126.9
t_{ann} = 6 h	12.9	2133.0	68.1	2213.9
As-Syn SMO	46.9	722.8	2145.0	2914.7
t_{ann} = 6 h	27.0	1066.0	173.8	1266.8
